# S100*β* Levels in CSF of Nonambulatory Dogs with Intervertebral Disk Disease Treated with Electroacupuncture

**DOI:** 10.1155/2013/549058

**Published:** 2013-12-29

**Authors:** Ayne Murata Hayashi, Ana Carolina Brandão Campos Fonseca Pinto, Silvia Renata Gaido Cortopassi, Valdecir Marvulle, Jessica Ruivo Maximino, Gerson Chadi, Julia Maria Matera

**Affiliations:** ^1^Department of Surgery, School of Veterinary Medicine and Animal Science, The University of São Paulo, 05508-270 São Paulo, SP, Brazil; ^2^Center of Mathematics, Computation and Cognition, The Federal University of ABC, 09210-170 Santo André, SA, Brazil; ^3^Department of Neurology, School of Medicine, The University of São Paulo, 01246-903 São Paulo, SP, Brazil

## Abstract

The aim of the study was to investigate S100*β* levels in the cerebrospinal fluid of nonambulatory dogs with intervertebral disk disease treated with electroacupuncture: 10 dogs with thoracolumbar disk extrusion graded 3 to 5 (EA group) and 7 dogs without neurologic dysfunction (control group). All dogs regained ambulation. S100*β* was detected by Western blot analysis where EA group dogs were evaluated at two time points (M1 = before EA and M2 = when the dogs return ambulation) and at one time point from control group. In EA group dogs M1-S100*β* levels were significantly higher than in control group. EA group dogs were divided into subgroups A (*n* = 7—early motor recovery; 6.7 ± 7.8 days) and B (*n* = 3—late motor recovery; 76 ± 17.0 days). M1-S100*β* levels were similar between subgroups A and B. However, M2-S100*β* levels were significantly higher in subgroup B than in subgroup A. An elevated S100*β* levels were observed in dogs with late motor recovery. S100*β* may be associated with neuroplasticity following spinal cord injuries with intervertebral disk extrusion. Further studies with larger numbers of subjects and control group with affected dogs are necessary to investigate the relationship between neurotrophic factors and electroacupuncture stimulation.

## 1. Introduction 

S100*β* is neurotrophic protein in the S100 family. This group of proteins is named “S100” due to solubility in 100% saturated ammonium sulfate solution [1] and is expressed only in vertebrates [2]. S100*β* is a low molecular weight protein (10 kDa) that is produced mainly by astrocytes and exerts paracrine and autocrine effects on neurons and glia [1]. Other calcium-binding proteins similar to S100*β* can buffer excess Ca^+2^ in central nervous system (CNS) cells and may aid in prevention of neuronal cell death [3].

S100*β* plays a role in the development of the brain, stimulates astroglial proliferation and maturation, and is neuroprotective [3]. It also promotes events possibly related to the plasticity of the spinal cord following injury, such as microtubule assembly and stimulation of neuritic outgrowth from the spinal cord and dorsal root ganglia [4]. Hence, the presence of astroglial S100*β* in regions of preserved tissue may be related to neuronal tropism and plasticity in the remaining spinal cord neurons and axons [3].

Despite the traditional use of acupuncture for treatment of different clinical conditions in China, access to research involving Chinese acupuncture is hampered by language constraints and studies involving the use of acupuncture for treatment of spinal cord injuries are hard to come by [5].

Nevertheless, acupuncture has been used for treatment of thoracolumbar intervertebral disk disease (IVDD) [6–8] and spinal cord injuries with paralysis and paresis [9] in dogs. Electroacupuncture can be more effective than phenylbutazone in treating chronic thoracolumbar pain in horses [10]. The use of electroacupuncture stimulation combined with standard Western medical for treatment of thoracolumbar IVDD in dogs has been evaluated and resulted in earlier return to ambulation compared with standard Western medical treatment alone [11]. Also, better results were obtained using electroacupuncture alone or in combination with decompressive surgery than with decompressive surgery alone [12]. However, none of these studies clarified how electroacupuncture influences neurological recovery in IVDD patients.

The purpose of this prospective study was to investigate the cerebrospinal fluid S100*β* levels in nonambulatory dogs with thoracolumbar disk extrusion before and after repeated electroacupuncture stimulation.

## 2. Materials and Methods

This research was approved by the Institutional Research Ethical Committee. Informed consent was obtained from the owners of all dogs prior to commencement of the study. The clinical study was performed at the Veterinary Hospital from School of Veterinary Medicine and Animal Science of the University of São Paulo. The experimental study (S100*β* research) was performed at the Experimental Neurosurgery Laboratory (LIM-45), Department of Neurology, from School of Medicine of the University of São Paulo. During 17 months, 66 dogs presenting with signs of thoracolumbar IVDD were evaluated, but only 10 were included in the study. Clinical, neurological, and radiographic evaluations were performed, apart from computed tomography (CT)-myelography. The day after the exam all dogs were treated with electroacupuncture alone (EA group, *n* = 10). The EA group consisted of 10 Dachshund dogs (6 males and 4 females). Mean age (mean ± SD) was 7 ± 2.4 years and mean body weight was 8.3 ± 2 kg. Cerebrospinal fluid (CSF) of mongrel dogs without neurological dysfunction that had been euthanized for reasons unrelated to the study (control group, *n* = 7) was collected for comparison.

The degree of neurological dysfunction of each dog was graded 1 to 5 as follows: grade 1 = no neurologic signs except pain associated with IVDD; grade 2 = conscious proprioceptive deficit and ambulatory paraparesis; grade 3 = nonambulatory paraparesis and deep pain perception; grade 4 = nonambulatory paraplegia and deep pain perception, with or without urinary dysfunction; and grade 5 = nonambulatory paraplegia and absence of deep pain perception, with or without urinary dysfunction.

During the first visit, inclusion and exclusion criteria were evaluated and the owners were informed about the implications related to the procedures involved in the study. *Inclusion Criteria*—nonambulatory dogs presenting with grade 3 to 5 neurological dysfunction that had never been submitted to surgery or EA and dogs diagnosed with thoracolumbar intervertebral extrusion by CT-myelography. *Exclusion Criteria*—dogs with skin ulcers, extensive wounds, or impaired hepatic and/or renal function that could not be submitted to general anesthesia for CT-myelography or CSF collection; dogs with unfavorable neurological outcomes presenting with clinical signs of ascending or descending myelomalacia; dogs that could not be brought in by their owners for weekly treatment.

### 2.1. CSF Collection

CSF collection for determination of S100*β* levels was performed under general anesthesia. The anesthetic plan included premedication with intramuscular meperidine 4 mg/kg, after 15 minutes, followed by intravenous propofol 6 mg/kg for induction of anesthesia and maintenance of general anesthesia. The same protocol was performed for CT-myelography exam except that isoflurane for maintenance of general anesthesia was added. Following clipping and skin preparation aseptic cerebellomedullary cysternal puncture was performed with a 22-gauge needle. The first few drops of CSF were discarded and a sample was collected into a sterile tube. CSF samples were stored at −70°C for later use. CSF samples were collected at two time points in dogs in the EA group (M1 = before EA and M2 = when the dogs were able to ambulate without assistance; M1-S100*β* levels and M2-S100*β* levels, resp.) and at one time point in dogs in the control group.

### 2.2. Response to Treatment and Functional Numeric Scale (FNS)

The time (days) required for a dog to be able to walk without assistance was recorded by the owner or one of the researchers in the study. Proprioception was evaluated at the hospital.

Improvements following EA treatment were evaluated by the same professional (AMH) by means of a FNS at three time points (M1 = before EA, M2 = when the dogs were able to ambulate without assistance, and M3 = last visit to the hospital). The FNS employed was the one described by Hayashi et al. [11]: ability to stand up (0 to 4); movement of pelvic limbs (0 to 4); degree of deep pain perception (0 to 4); urinary control (0 to 4); ability to walk (0 to 4); movement of the tail (0 to 3). A score for evaluation of proprioception was also included: lack of conscious proprioception (0), moderately reduced conscious proprioception in at least one limb (1), mildly reduced conscious proprioception in at least one limb (2), and normal conscious proprioception (3). The sum of all scores ranged from 0 (worst possible) to 26 (absence of neurological signs). Tail movement was classified according to observations by the owner (e.g., if the dog moved the tail as the owner approached, this was considered voluntary movement). Successful outcome was defined as the ability of the dog to walk without assistance or/and return of deep pain perception.

### 2.3. Scores (0–3) of Extradural Spinal Cord Compression and Presence of Calcified Material in the Vertebral Canal and Respective CT-Myelography Features

Lack of extradural spinal cord compression or calcified material in the vertebral canal (0); mild extradural spinal cord compression with displacement or presence of calcified material causing less than 25% vertical compression of the vertebral canal (1); moderate extradural spinal cord compression with flattening or presence of calcified material causing 50% vertical compression of the vertebral canal (2); obstruction of more than 50% of the vertebral canal space by calcified material (3).

### 2.4. Scores (0–4) of Number of Intervertebral Disk Spaces Affected by Extradural Spinal Cord Compression

Absence of lesions (0); one disk space affected (1); two disk spaces affected (one main lesion and a second, mild one) (2); three disk spaces affected (one main lesion and two mild ones) (3); more than three disk spaces affected (one main lesion and more than two mild ones) (4).

### 2.5. Electroacupuncture

**Figure 1:**
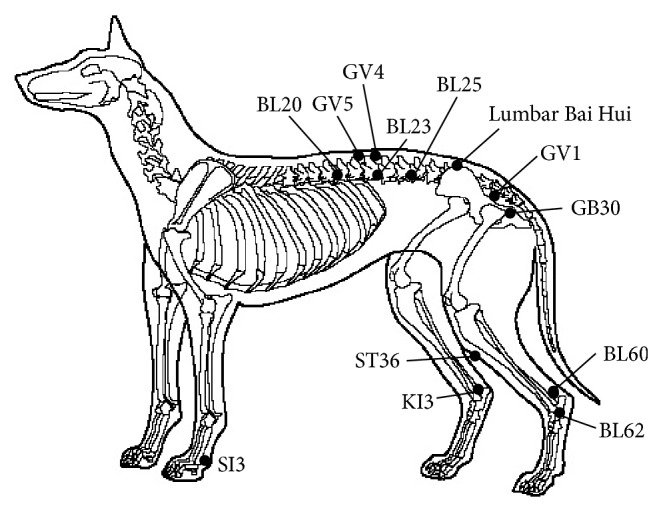
Locations of acupuncture points: SI 3—lateral side of the fifth metacarpophalangeal joint, proximal to the head of the fifth metacarpal bone, BL20—lateral to the caudal border of the spinous process of the twelfth thoracic vertebra, BL23—lateral to the caudal border of the spinous process of the second lumbar vertebra, BL25—lateral to the caudal border of the spinous process of the fifth lumbar vertebra, ST36—on the craniolateral aspect of the pelvic limb, three-sixteenths of the distance from the junction between the patella and patellar ligament to the cranial tarsus, about one digit breadth lateral to the tibial crest (lateral portion of the cranial tibial muscle), KI3—between the malleoli and the talus, BL60—opposite from KI3, BL62—in the depression distal to the lateral malleolus of the fibula, GB30—in the depression midway between the greater trochanter of the femur and the tuber ischii, GV1—in the depression between the anus and base of the tail, GV4—on the dorsal midline, between the dorsal spinous processes of the second and third lumbar vertebrae, GV5—on the dorsal midline, between the dorsal spinous processes of the thirteenth thoracic and first lumbar vertebrae, and Lumbar Bai Hui—on the dorsal midline, in the depression at the lumbosacral junction.

Percutaneous electroacupuncture was performed using an EA device (model DS 100 CB, Sikuro, Sikuro Sistemas e Equipamentos Elétricos Ltda, Rio de Janeiro, Brazil) and acupuncture needles with the size of 0.25 × 25 mm (Cloud & Dragon, WuJiang City Cloud & Dragon Medical Device Co., Ltd., Jiangsu, China). Selection and location of the acupuncture points were based on the author's clinical experience [11] and the veterinary literature [13–15]. Different governing vessel (GV) points were used depending on location of the extrusion lesion. Acupuncture points (Figure 1) selected according to traditional Chinese medicine and related neuroanatomical structures [13, 14] were as follows: small intestine (SI) 3—fifth abaxial dorsal digital nerve; bladder (BL) 62, 20, and 23—caudal cutaneous sural nerve, dorsal cutaneous branch of the twelfth thoracic nerve, dorsal cutaneous branch of the second lumbar spinal nerve, respectively; stomach (ST) 36—branches from the saphenous nerve, and deeply to the point there is the peroneal nerve; kidney (KI) 3 (saphenous nerve) transfixed with BL60 (caudal cutaneous sural nerve); GV1 (ventral branches of the sacral and coccygeal nerves) and local GV points and lumbar Bai Hui (medial branch of the seventh lumbar nerve). In some dogs BL25, related to the dorsal cutaneous branch of the fifth lumbar spinal nerve, was added as local point; gallbladder (GB) 30, related to gluteus cranial nerve, lateral cutaneous femoral nerve, and cutaneous branches of the sacral nerves and deeply there is the sciatic nerve, was used only in dogs that did not allow access to GV1. All electroacupuncture treatments were performed by a small animal veterinarian certified in veterinary acupuncture (by International Veterinary Acupuncture Society, IVAS). Pairs of acupuncture points on the same side of the body were connected by an electrode to form a set, which was then subjected to alternating currents of 3 Hz and 100 Hz for 3 seconds each over a period of 20 minutes [11]. Voltage was increased until muscle twitching was observed. The sets of acupuncture points employed were BL20 or BL25 and BL23, lumbar Bai Hui and GV1 or GB30 (right side), and ST36 and KI3 transfixed to BL60 (both sides). Electroacupuncture stimulation was performed in all sets at the same time. The remaining points were stimulated by needle insertion only. All dogs received electroacupuncture treatments once per week for 3 weeks or until the dogs were able to walk without assistance and improvement of neurological deficits.

### 2.6. Determination of S100*β* Levels by Western Blot Analysis

CSF S100*β* protein levels were assessed by semiquantitative Western blot analysis [16]. Protein concentrations in CSF samples were determined according to the method described by Bradford [17]. CSF samples (50 *μ*g protein) were separated by 20% sodium dodecyl sulfate (SDS)—polyacrylamide (Bio-Rad, Bio-Rad Laboratories Incorporation) gel electrophoresis. Proteins were transferred onto nitrocellulose membranes (Bio-Rad, Bio-Rad Laboratories Incorporation) for 40 minutes in 100 V. Following 30 minutes blocking with 3% bovine serum albumin (BSA) in Tris-buffered saline tween (TBS-T) membranes were incubated overnight with mouse antibody against S100*β* (Abcam, Abcam plc.) at 1 : 800 in 5% milk/TBS-T. Anti-S100*β* antibody ab11178 (full length native purified bovine protein that recognizes S100*α* and S100*β* in different mammal species, Abcam, Abcam plc.) was used. Following incubation membranes were washed twice in TBS-T for 10 minutes and incubated at room temperature for 1 hour with a 1 : 6000 dilution of anti-mouse IgG-ECL conjugated secondary antibody (GE, GE Healthcare UK limited). Blots were then washed twice with TBS-T and once with TBS. After the final washes membranes were incubated with Western Lightning Chemiluminescence Reagent Plus (PerkinElmer, PerkinElmer Inc.) for 1 minute. Membranes were exposed to X-ray film (Hyperfilm ECL, Amersham Biosciences) for imaging and visualization of protein bands. S100*β* levels were quantified by densitometry using a computer-assisted image analyser and the software developed by Imaging Research (Imaging Research Inc., Brock University).

### 2.7. Statistical Analysis

The method Kolmogorov and Smirnov was used to test normality of the variables. The sample size (*n* = 7) was calculated with level of significance of 1% (*α* = 0.01) and power of 90% (1 − *β* = 0.90). The parametric one-way analysis of variance (ANOVA) and the Bonferroni posttest for multiple comparisons were used to compare the differences between FNSs at each of the 3 time points. Parametric Student's *t*-test for repeated measures (variables) was used to compare EA group M1 and M2-S100*β* levels, and parametric Student's *t*-test for independent measures (variables) was used to compare these with control group S100*β* levels. The Pearson correlation was used to investigate the correlation between EA group M1-S100*β* levels and quantitative variables (duration of clinical signs prior to treatment, age, time to recovery of ambulation, body weight, and length of extruded disk material (mm)). To verify the associations between EA group M1-S100*β* levels and qualitative variables such as lesion grade (3 to 5), extradural compression, number of lesions, and FNS scores, data were grouped according to different grades or scores and compared using ANOVA or Student's *t*-test for independent variables. The Pearson correlation was used to investigate the correlation between EA group M2-S100*β* levels and quantitative variables (duration of clinical signs prior to treatment, time to recovery of ambulation, and length of extruded disk material (mm)). The level of significance was set at 5% (*P* < 0.05) for all statistical tests.

## 3. Results 

### 3.1. Clinical Data

**Figure 2:**
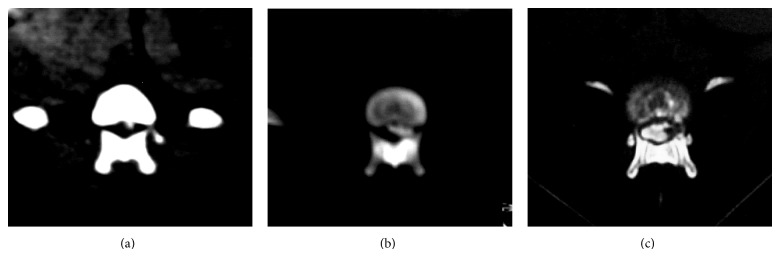
Transverse computed tomography images following myelography and representative extradural compression scores for comparisons. Dogs with extradural compression scores: (a) score 1 at T11-T12, (b) score 2 at T11-T12, and (c) score 3 at L1.

Duration of clinical signs prior to treatment was 30.8 ± 14.3 days. Neurological dysfunction was graded 3 (2 dogs), 4 (6 dogs), and 5 (2 dogs). Extradural compression scores based on CT-myelography (Figure 2) were 1 (*n* = 1), 2 (*n* = 6), and 3 (*n* = 3). Scores of number of lesions were 1 (*n* = 2), 2 (*n* = 4), 3 (*n* = 2), and 4 (*n* = 2). Length of extruded disk material (mean ± SD) was 7.2 ± 2.6 mm. The incidence of disk extrusion was 30% at T11-12, 30% at T12-13, 10% at T13-L1, and 30% at L1-2 with extrusion affecting either the left or the right side (50% each). Multiple lesions were found in 80% of the dogs (8 out of 10) with extradural extrusions found at L3-4 (2) and L4-5 (1). Mild extradural compression was observed at T11-12 (4), T12-13 (3), T13-L1 (2), and C2-3 (1).

### 3.2. Recovery of Ambulation

All dogs recovered motor function. One dog did not regain deep pain perception but regained spinal walking reflex and was able to walk without assistance. Mean time for recovery of unassisted ambulation, even if intermittent, was 27.5 ± 35 (mean ± SD) days. During the reevaluation period dogs in the EA group were divided into subgroups A (*n* = 7—dogs that regained ambulation before 30 days; 6.7 ± 7.8 days) and B (*n* = 3—dogs that regained ambulation after 30 days; 76 ± 17.0 days). Clinical data (mean ± SD) did not differ significantly between subgroups A and B dogs. Age was 6.4 ± 1.8 and 7.0 ± 4.4 years (*P* = 0.27); body weight was 8.4 ± 2.1 and 7.9 ± 2.1 kg (*P* = 0.75); duration of clinical signs prior to EA treatment was 30.7 ± 16.1 and 31.0 ± 11.8 days (*P* = 0.97); grade of neurological dysfunction was 3.7 ± 0.8 and 4.3 ± 0.6 (*P* = 0.24); FNS score was 11.3 ± 3.6 and 7.0 ± 4.4 (*P* = 0.14); extradural compression score was 2.3 ± 0.8 and 2.0 ± 0.0 (*P* = 0.35); number of extrusions score was 2.0 ± 0.8 and 3.3 ± 1.2 (*P* = 0.06); and length of extruded disk material was 6.9 ± 3.0 and 8.0 ± 2.0 mm (*P* = 0.57), respectively. However, time to recovery of ambulation differed significantly (*P* < 0.001) between subgroups A (6.7 ± 3.0 days) and B (76.0 ± 17.1 days). Subgroup A dogs received 1.8 ± 1.0 electroacupuncture treatments before recovering the ability to ambulate and 7.4 ± 1.7 sessions in total, while subgroup B dogs required 11.3 ± 2.3 electroacupuncture treatments to recover the ability to ambulate and received 20.3 ± 4.7 sessions in total.

Age and weight had no correlation with time to recovery of ambulation of EA group, respectively, *r* ^2^ = 0.20; *r* = 0.44; *P* = 0.19 and *r* ^2^ = 0.03; *r* = 0.19; *P* = 0.58. Duration of clinical signs prior to EA treatment and length of extruded disk material had no correlation with time to recovery of ambulation, respectively, *r* ^2^ = 0.01; *r* = 0.13; *P* = 0.71 and *r* ^2^ = 0.06; *r* = 0.25; *P* = 0.47. Extradural compression score and number of extrusions score were not correlated with time to recovery of ambulation, respectively, *r* ^2^ = 0.04; *r* = 0.22; *P* = 0.54 and *r* ^2^ = 0.27; *r* = 0.52; *P* = 0.11.

### 3.3. Evaluation of FNS Scores

Following EA, EA group FNS scores increased significantly between time points (M1, M2, and M3, resp.). However, when the subgroups were considered FNS scores increased significantly between M1 and M2, with no significant differences between M2 and M3 (Table 1).

### 3.4. S100*β* Levels

Data on S100*β* levels are displayed in Table 2. In EA group dogs M1-S100*β* levels did not differ from M2-S100*β* levels (*P* = 0.40). However, M1-S100*β* levels were significantly higher than S100*β* levels in control group dogs (*P* = 0.03), with no significant differences between M2-S100*β* levels and S100*β* levels in control group dogs (*P* = 0.11). In subgroup A dogs M1-S100*β* levels were higher (*P* = 0.01) than M2-S100*β* levels and S100*β* levels in control group dogs, but M2-S100*β* levels did not differ significantly from S100*β* levels in control group dogs (*P* = 0.42). In subgroup B dogs M2-S100*β* levels were significantly higher (*P* = 0.03) than M1-S100*β* levels and S100*β* levels in control group dogs, but M1-S100*β* levels did not differ significantly from S100*β* levels in control group dogs (*P* = 0.75). M1-S100*β* levels did not differ significantly (*P* = 0.06) between dogs in subgroups A and B. However, M2-S100*β* levels were significantly higher in subgroup B than in subgroup A dogs (*P* = 0.03).

M1-S100*β* levels were not correlated with age (*r* ^2^ = 0.20; *r* = −0.45; *P* = 0.18), body weight (*r* ^2^ = 0.02; *r* = 0.16; *P* = 0.65), duration of clinical signs prior to treatment (*r* ^2^ = 0.00; *r* = −0.07; *P* = 0.82), speed of recovery of ambulation (*r* ^2^ = 0.25; *r* = −0.50; *P* = 0.13), or length of extruded disk material (*r* ^2^ = 0.03; *r* = −0.18; *P* = 0.60). M1-S100*β* levels did not differ significantly according to grade of neurological dysfunction (*P* = 0.93), grouped scores of extradural compression (*P* = 0.77), number of lesions (*P* = 0.52), or FNS findings (*P* = 0.77).

M2-S100*β* levels were not correlated with duration of clinical signs prior to treatment (*r* ^2^ = 0.03; *r* = −0.17; *P* = 0.62), length of extruded disk material (*r* ^2^ = 0.01; *r* = −0.12; *P* = 0.72), or speed of recovery of ambulation (*r* ^2^ = 0.38; *r* = 0.62; *P* = 0.05).

## 4. Discussion

Thoracolumbar intervertebral disk extrusion is a common cause of neurologic dysfunction in dogs. Clinical signs can range from signs of pain or spinal hyperesthesia to pelvic limb paresis or paralysis with or without deep pain perception and urinary retention [18]. In the present study all dogs recovered ambulation without assistance, including the one that regained spinal reflex walking. This is in agreement with results of a previous study [11] where electroacupuncture combined with Western treatment anticipated the recovery of ambulation in dogs with grade 3 to 4 neurological dysfunction (10.1 days) when compared with dogs receiving Western treatment alone (20.8 days).

The spinal reflex walking is noted in paraplegic animals that regain a complex and reciprocal alternating pattern of hindlimb movements. The centers responsible for coordination of complex gaits—called central pattern generators (CPGs)—are more complex than simple spinal reflexes that enable a specific sensory input to activate a single-phased motor response. Repetition of task-specific movements, including part-weight-supported walking, can reactivate these CPGs [19]. Electroacupuncture may have the ability to “train” this pattern given that it is considered a sensory stimulation [20] and can promote muscle contraction responses at many motor points that are also acupuncture points [21]. These factors may have contributed to the results obtained in this and previous studies involving electroacupuncture [11, 12] and may have accelerated reactivation of CPGs.

The results of this study point to individual differences in recovery following spinal cord injury. Investigating changes in white matter following spinal cord injuries, Smith and Jeffery [22] found that the complexity of naturally occurring injuries resulted in individual variations. In the present study, dogs of the same chondrodystrophic breed had different recovery speeds. This may be related to differences in injury type and severity and/or individual responses to initial trauma. Higher M1-S100*β* CSF levels in dogs with early motor recovery suggest potential self-regulation in glial cells. Lower M1-S100*β* CSF levels in dogs with late motor rehabilitation indicate that glial cell related compensatory mechanisms in response to injury were not stimulated until approximately 4 weeks following injury, although the increase in S100*β* levels during the EA treatment period suggests potential modulation and compensatory responses to initial injury. Our results agree with Cunha et al. [3] who observed increases in S100*β* and fibroblastic growth factor-2 in reactive astrocytes but not in inactive microglia following spinal cord injury in adult rats. This may have triggered a recovery response close to the site of injury and was possibly related to motor recovery in these animals.

Differentiation and migration of astroglial cells in the spinal cord following injury to the dorsal surface have been reported. Newly formed astrocytes were noted first at the dorsal surface of degenerating white and grey matter in the spinal cord with subsequent ventral migration and differentiation into white or grey matter astrocytes. Astroglial precursors were observed within 30 hours of injury, decreased after 1 week, and reached undetectable levels within 3 months [23]. CSF S100*β* levels are directly related to glial cell activity. The results of this study reflect the findings by Kozlova [23] and suggest the early motor recovery observed in most dogs may result from increased astroglial response given that S100*β* levels were high prior to treatment and decreased following recovery of ambulation. Hence S100*β* levels are possibly self-regulated in response to plasticity and neural tissue repair. Had CSF collection been performed prior to recovery of ambulation in this group of dogs, increased S100*β* levels might have been detected following EA.

S100*β* levels were associated with the pathophysiology of neuropathic pain in an experimental model where high levels were observed within 14 days of spinal cord injury [24]. However, clinical signs of pain were not present at any of the time points analyzed in this study so an association between pain and S100*β* levels was not possible.

S100*β* serum [25, 26] and CSF [27] levels are considered one of the markers for spinal cord tissue injury. High S100*β* serum levels were observed within 6 hours of spinal cord injury, with great variability in 24 hours [25]. High peaks of S100*β* were also observed up to 72 hours following spinal cord injury, with return to normal levels in 6 days. A similar pattern was documented following lumbar plexus avulsion, although S100*β* levels were lower in this case than with spinal cord injury [26]. S100*β* levels could not be associated with acute injury in this study given that the first measurements were performed 4 weeks following injury, in average. Also if the lumbar CSF was included in the present study we could have had other findings because it reflects the spinal cord disease changes as it takes places caudal to the main lesions of the affected dogs. Another critical observation is that our control group consisted of 7 mongrel dogs and the EA group consisted of chondrodystrophic dogs. Future studies must include affected chondrodystrophic dogs as control groups so it will be possible to compare the S100*β* variations more closely.

Secondary events triggered by spinal cord injury have the ability to induce cell death but they also aid in function recovery by increasing the release of local growth factors, which in turn stimulate the proliferation of endogenous progenitors. Such endogenous recovery mechanisms support the chronic repopulation of the injured spinal cord by glial cells. However this is devoid of significance if normal function is not regained [28]. In this study the later motor recovery of some dogs suggests EA treatment may have the ability to modulate endogenous proliferation of progenitor cells and late recovery of glial cell density.

Complete recovery from spinal cord injuries can only be achieved by means of multiple treatment modalities. Treatment goals are maintenance of minimal degree of injury, preservation and increase of residual function, and promotion of regeneration [19]. Interventions at the neuronal microenvironment level are required to promote regeneration and protection of injured spinal cord neurons [29]. The positive results following EA application in dogs in this study suggest it is a reasonable treatment even for dogs with severe neurological injuries. We believe EA could be used to restore function in patients presenting with concussion and hematoma not only due to stimulation of cells in the injured tissue but also in adjacent areas that become reactive and trigger neuronal plasticity, although this had not been experimentally confirmed to date.

Neurological disorders such as paralysis following intervertebral disk extrusion in dogs involve neuronal injury and impaired neuronal propagation [30]. For cell regeneration to occur electrical transmission has to be maintained or established through existing and newly formed neural elements [19]. The purpose of acupuncture is to rectify electrical impairments reducing electrical resistance and enhancing electrical activity in injured tissues [9]. Therefore, acupuncture promotes healing and axonal regrowth [31]. Although the mechanisms involved in neuronal regeneration by EA are not clearly understood [32], there is a connection between stimulation of peripheral acupuncture points and central neural pathways [33]. EA can induce neural activation and thus has potential clinical applications.

Acupuncture treatment can signal surviving neurons to produce neuronal growth factors that maintain and rehabilitate normal neuronal tracts [30]. To our knowledge investigation of S100*β* protein levels in CSF of nonambulatory dogs has not been reported to date. The results of this study suggest that the elevation of S100*β* levels following EA may reflect this signaling mechanism, with triggering of different events or release of additional neurotrophic factors that promote regeneration. Ethical implications precluded the inclusion of a nontreated group of dogs affected with disk extrusion or the use of a *sham* acupuncture group for comparison of clinical signs and CFS S100*β* levels in this study. An additional group with CSF of dogs with thoracolumbar disk extrusion without electroacupuncture should be more accurate for the study and could have elucidated the S100*β* modulation after electroacupuncture. Also a blind evaluation of the groups is preferable but it must involve more researchers to take part in the study. Further experimental studies are required to investigate additional neurotrophic factors involved in this modality of neuronal stimulation. The exact mechanisms by which EA stimulates motor rehabilitation and which degrees of spinal cord injury are associated with S100*β* modulation remain to be elucidated.

## 5. Conclusions

An elevated S100*β* levels were observed in dogs with late motor recovery. S100*β* may be associated with neuroplasticity following spinal cord injuries after intervertebral disk extrusion. Only experimental studies could confirm this finding at the spinal cord after the lesion. As other researchers previously have found, electroacupuncture can be an integrative modality to help motor recovery in dogs with IVDD.

## Figures and Tables

**Table 1:** Functional numeric scale (mean ± SD) at different time points, per group.

Time point	M1	M2	M3
EA group (*n* = 10)	10.0 ± 4.1^a^	20.0 ± 4.6^b^	22.5 ± 3.8^c^
Subgroup A (*n* = 7)	11.2 ± 0.6^a,A^	21.5 ± 1.2^b^	23.8 ± 2.1^b^
Subgroup B (*n* = 3)	7.0 ± 4.3^a,A^	16.3 ± 8.0^b^	19.6 ± 5.7^b^

^a–c^Means with different superscripts within the same row are significantly different (*P* < 0.05). ^A-B^Means with different superscripts within the same column are significantly different (*P* < 0.05). M1 = first evaluation prior to EA treatment; M2 = when the dogs return ambulation without assistance; M3 = last evaluation. EA group = treated by electroacupuncture and Subgroups A and B = early and late motor recovery, respectively.

**Table 2:** S100*β* levels (mean ± SD), two different time points—electroacupuncture groups compared with control group.

Time point	M1	M2	Control group
EA group (*n* = 10)	0.1412 ± 0.01^a^	0.1345 ± 0.01^a,c^	0.1222 ± 0.01^b,c^
Subgroup A (*n* = 7)	0.1481 ± 0.01^a,A^	0.1281 ± 0.01^b,A^	0.1222 ± 0.01^b^
Subgroup B (*n* = 3)	0.1252 ± 0.01^a,A^	0.1495 ± 0.01^b,B^	0.1222 ± 0.01^a^

^a–c^Means with different superscripts within the same row are significantly different (*P* < 0.05). ^A-B^Means with different superscripts within the same column are significantly different (*P* < 0.05). CSF = cerebrospinal fluid. M1 = first CFS collection prior to EA treatment; M2 = second CSF collection, when the dogs return ambulation. EA group = treated by electroacupuncture and subgroups A and B = early and late motor recovery, respectively. Control group = dogs without neurological signs.

## Abbreviations

CSF: Cerebrospinal fluid
CT: Computed tomography
EA: Electroacupuncture
FNS: Functional numeric scale
IVDD: Intervertebral disk disease.
